# Neuropsychiatric and sleep study in autosomal dominant dopa-responsive dystonia

**DOI:** 10.1016/j.ymgmr.2022.100870

**Published:** 2022-04-18

**Authors:** Ailton C. Alves Júnior, Maurício V. Daker, Alexei M.C. Machado, Alan S. Luna, Dirceu C. Valladares Neto, Eugenia R. Valadares

**Affiliations:** a Federal University of Minas Gerais, School of Medicine, Graduate Program in Child and Adolescent Health, Av. Prof. Alfredo Balena, 190 – Sala 503, Belo Horizonte Cep: 30130-100, Minas Gerais, Brazil; b Federal University of Minas Gerais, School of Medicine, Department of Mental Health, Av. Prof. Alfredo Balena, 190 – Sala 235, Belo Horizonte Cep: 30130-100, Minas Gerais, Brazil; c Federal University of Minas Gerais, School of Medicine, Department of Anatomy and Image, Av. Prof. Alfredo Balena, 190 – Sala 179, Belo Horizonte Cep: 30130-100, Minas Gerais, Brazil; d Federal University of Minas Gerais, School of Medicine, Graduate Program in Child and Adolescent Health. Av. Prof. Alfredo Balena, 190 – Sala 503, Belo Horizonte Cep: 30130-100, Minas Gerais, Brazil; e Clínica do Sono, Alameda do Ingá, 780, Vale do Sereno, Nova Lima Cep: 34006-042, Minas Gerais, Brazil; f Federal University of Minas Gerais, School of Medicine, Graduate Program in Child and Adolescent Health, Av. Prof. Alfredo Balena, 190 – Sala 503, Belo Horizonte Cep: 30130-100, Minas Gerais, Brazil; g Pontifical Catholic University of Minas Gerais, Graduate Program in Informatics, Av. Itaú, 525, Dom Cabral, Belo Horizonte Cep: 30535012, Minas Gerais, Brazil

**Keywords:** Dystonia, Dopa-responsive, Autosomal dominant familial dystonia, Autosomal dominant GPCH1 deficiency, Dystonic disorders, Sleep-wake disorders, Mental disorders, ADHD CHD, Attention deficit/hyperactivity disorder in childhood., BDI-II, Beck Depression Inventory – II., BH4, Tetrahydrobiopterin cofactor., DRD, Dopa-responsive dystonia., DYT/PARK-*GCH1*, Dopa-responsive dystonia syndrome caused by GTP cyclohydrolase-1 gene variation., DYT5a, Autosomal dominant Segawa syndrome., ESS, Epworth Sleepiness Scale., GAD, Generalized Anxiety Disorder., *GCH1*, The official gene symbol approved by the HGNC for GTP cyclohydrolase-1 gene., GTP Cyclohydrolase-1, guanosine triphosphate cyclohydrolase-1 enzyme., HGNC 4193, HGNC ID for gene of the GTP cyclohydrolase-1., L-Dopa, Levodopa., MDE, Major depressive episode., MDE PAST, Past major depressive episode., MINI/MINI PLUS, Mini International Neuropsychiatric Interview., NMS, non-motor symptoms, NSG, Non-Segawa group., PSQI, Pittsburgh Sleep Quality Index., SG, Segawa group., WHOQOL-BREF, World Health Organization Quality of Life - BREF instrument., WMT-2, Vienna Matrix Test 2.

## Abstract

**Introduction:**

Although the diurnal fluctuation of motor dysfunction, reversible with small doses of dopamine, is a cornerstone for the phenotype of the autosomal dominant Segawa syndrome, the non-motor symptoms of this neurotransmitter deficiency have still received limited attention.

**Objective:**

This study aims to evaluate non-motor symptoms of this dopa-responsive dystonia through an intrafamilial comparative cross-sectional study.

**Methods:**

Seventeen individuals with a c.IVS5 + 3insT (c.626 + 3insT) variation in the GTP cyclohydrolase-1 gene (*GCH1*, HGNC: 4193) and 34 intrafamilial controls were studied using the Beck Depression Inventory-II, the Wiener Matrizen Test 2, the Epworth Sleepiness Scale, the Pittsburgh Sleep Quality Index, the MINI/MINI PLUS Questionnaires, the World Health Organization Quality of Life – BREF Instrument and a drug use assessment questionnaire.

**Results:**

No significant difference was found between the groups in the prevalence of sleep disorders and in cognitive function. Nevertheless, generalized anxiety disorder (*p* = 0.050) and attention-deficit/hyperactivity disorder in childhood (*p* = 0.011) were observed only in individuals without the molecular variation. The group with the *GCH1* variation presented a worse perception about how safe they feel in their daily lives (*p* = 0.034), less satisfaction with themselves (*p* = 0.049) and with their relationships (*p* = 0.029), and a higher prevalence of past major depressive episodes before use of L-Dopa (*p* = 0.046).

**Conclusion:**

Low dopamine could have been protective against generalized anxiety disorder and attention-deficit/hyperactivity disorder in childhood in Segawa group individuals. The prevalence of depression was higher in individuals with the molecular variant prior to the L-Dopa treatment. Considering it, the penetrance estimates for the variant carriers increased from 58.8% to up to 88% in this large studied family. Additionally, neuropsychiatric tests of all individuals with a molecular diagnosis in an affected family are a valuable instrument for its clinical management.

## Highlights

• Depression can be a non-motor symptom of autosomal dominant DYT/PARK-*GCH1*.
• Variable expressivity and penetrance can be considered high in this syndrome.
• Relatives without dystonia should also be investigated for the genetic variation.

## 1 Introduction

The dopa-responsive dystonia (DRD) caused by the autosomal dominant variant in the guanosine triphosphate cyclohydrolase-1 gene (currently identified as autosomal dominant DYT/PARK-*GCH1*) is mainly characterized by the appearance of posture and motor locomotion disturbances with marked diurnal variation in childhood or adolescence and a simultaneous or late occurrence of parkinsonism [[1], [2], [3]]. Also known as Segawa syndrome or DYT5a dystonia (OMIM # 128230), its motor symptoms treated with low dosages of levodopa (L-Dopa) have an excellent response in a period of a few weeks [2,4]. It is a rare disease, with around 1–9 cases per million people [5,6].

The variations in the gene of the GTP cyclohydrolase-1 (guanosine triphosphate cyclohydrolase-1), EC 3.5.4.16, symbol *GCH1*, HGNC: 4193, located at the chromosome 14q22.1-q22.2, lead to a deficiency of this enzyme, one of the responsible for the conversion of GTP to tetrahydrobiopterin (BH4). BH4 is a cofactor for the aromatic amino acid hydroxylases (phenylalanine-4-hydroxylase, tyrosine-3-hydrolase, and tryptophan-5-hydroxylase) to produce tyrosine, dopamine, and serotonin.

The autosomal dominant DYT/PARK-*GCH1* has variable expressivity and, based on a recent systematic review, asymptomatic individuals totaled approximately 25% (151 of 639 mutation carriers), resulting indirectly in penetrance of 75%; however, these authors argue that the population penetrance can be lower [7].

Nowadays, the autosomal dominant DYT/PARK-*GCH1* is still under-diagnosed, which is also associated with the fact that many signs and symptoms are not severe enough to motivate the patients to seek clinical assistance [[8], [9], [10]].

Equally, although the role of altered dopamine metabolism in motor dysfunction in this DRD is known and reversible, the consequences of synthesis deficiencies of this and other neurotransmitters involved in the pathogenesis of possible non-motor symptoms (NMS) in this disease, like serotonin and noradrenaline, have still received limited attention. Antelmi et al. (2015) state that, given the numerous functions of the BH4 cofactor, it is also reasonable to assume that in autosomal dominant DYT/PARK-*GCH1*, other systems, in addition to the motor, would be impaired [11]. Also, for these and other authors, such as Tadic et al. (2012) and Timmers et al. (2017), NMS may be being neglected, and/or very few and contrasting data are currently available on the subject [[11], [12], [13], [14], [15], [16]].

This study aims to improve the knowledge about NMS of autosomal dominant DYT/PARK-*GCH1* by comparing the prevalence of some neuropsychiatric and sleep disorders in individuals with this DRD to their not molecularly affected relatives. The penetrance of this disease may be higher than published previously.

## 2 Materials and methods

The sample of this comparative cross-sectional study with case and control groups was initially composed of 63 individuals taken from a single Brazilian family. Nineteen members (30.2%) had a c.IVS5 + 3insT (c.626 + 3insT) variation in the splicing regulatory element of the *GCH1* gene, according to a previous genetic study [17]. Among the carriers of the variation, 15 (78.9%) were females, 9 of them with motor symptoms, and 4 (21.1%) males, only one of them with motor symptoms [17]. Since that initial study, four individuals died (2 carriers – 1 male and one female – and two non-carriers). Additionally, the other three non-carrier individuals were excluded from the sample because they moved to distant locations. These facts represented an exclusion of 7 of the 63 possible study participants.

After enrollment of the 56 individuals (17 carriers and 39 non-carriers), five non-carriers did not complete the assessment and were excluded. Thus, the case group was composed of 17 carriers and the control group of 34 non-carriers. The Ethics Committee of the Federal University of Minas Gerais approved the study (number 06803312.8.0000.5149), performed between March 2016 and November 2017. Signed informed consent was obtained from all participants.

Only assessment instruments in the Brazilian Portuguese language were considered. The World Health Organization Quality of Life – BREF instrument (WHOQOL-BREF) was used to analyze the quality of life [18]. A neuropsychiatric evaluation was performed by two mental-health professionals who applied the Mini International Neuropsychiatric Interview (MINI) version 5.0 and the adapted W module of the MINI PLUS 5.0.0 (June 2001) for the diagnostic investigation of Attention-Deficit/Hyperactivity Disorder [19]. In order to investigate major depressive episodes, questionnaires inquired about current, past, and recurrent events. The mental-health professionals were unaware of the interviewees' condition regarding the presence of the variation. The Beck Depression Inventory-II (BDI-II) was selected to detect depressive symptoms and to measure the severity of these episodes, using a lower cut-off threshold to increase sensitivity [20,21]. The non-verbal Vienna Matrix Test 2 (WMT-2) was used for the intellectual evaluation of the participants with the Table of Standards for 14–69 years, free time, regardless of education level and the classification of the percentile for the interpretation of the gross results [22].

For the subjective evaluation of sleep disorders, the Epworth Sleepiness Scale (ESS) and the Pittsburgh Sleep Quality Index (PSQI) were applied [23,24]. The higher the total score was in ESS (0–24), the higher was the level of daytime sleepiness of the self-assessment, and the scores above 10 suggested the diagnosis of excessive daytime sleepiness [25]. The PSQI, also self-applicable, stratified the individual as a “good sleeper” (if overall score up to 5) or “bad sleeper” (overall score greater than 5), through 7 components assessed in the previous month [24].

A brief structured assessment of the history of chronic medication use was also applied to the participants, mainly specifying the use of L-Dopa, antidepressants, anxiolytics, and other psychoactive drugs.

For the WHOQOL-BREF and the assessment of medication use, 51 participants answered the questionnaires. The Non-Segawa group (NSG) was composed of 23 females and 11 males (*N* = 34), with a mean(s.d.) age of 41.0(17.5) years, and the called Segawa group (SG) was composed of 14 females and three males (*N* = 17), with a mean(s.d.) age of 48.9(18.4) years. For the MINI, BDI-II, ESS, and PSQI instruments, 38 participants answered the questionnaires: 12 females and nine males for the NSG (*N* = 21), with a mean(s.d.) age of 43.2(19.4) years; the SG kept the same previous member composition. For the WMT-2 instrument, participants older than 69 years were excluded so that the sample (*N* = 31) was composed of 10 females and seven males in the NSG, with a mean(s.d.) age of 39.9(15.8) years and 12 females and two males in the SG, with a mean(s.d.) age of 44.4(17.0) years.

Statistical analysis was performed using the R software version 3.3.3. Considering that the size of the samples was too small to characterize their distribution, the nonparametric tests of Wilcoxon and Wilcoxon adjusted for age and sex were preferred to Student's *t*-test in the case of numeric variables, and Fisher's exact test was applied to categorical data. Although the Segawa and control samples were considerably matched for sex and age, these covariates were corrected using linear regression, as they could act as confounders in the analysis of depression and quality of life. A Type I error level α = 0.05 was considered for all analyses.

## 3 Results

### 3.1 Assessment of quality of life

The overall quality of life expressed as a 0–100 score presented a mean (s.d.) of 70.6 (19.9) and 72.1 (15.0), respectively, for the NSG and SG, with no significant difference between groups after adjustment (*p* = 0.425). Nevertheless, the Wilcoxon test adjusted for age and sex revealed statistically significant differences between these groups in the following variables that were measured in a scale from 1 to 5 (Table 1):

(a) Question 3 _ “In the past two weeks, to what extent did you feel that (physical) pain prevented you from doing what you needed to do?” although both mean scores closer to the response option “a little”;
(b) Question 4 _ “In the past two weeks, how much did you need any medical treatment to execute functions in your daily life?” whose mean score in SG was closer to the response option “a moderate amount” and in NSG closer to the option “a little”;
(c) Question 8 _ “In the past two weeks, how safe did you feel in your daily life?” whose mean score in SG was closer to the response option “a moderate amount” and in NSG closer to the option “very much”;
(d) Question 19 _ “In the past two weeks, how satisfied were you with yourself?” whose mean score in SG was equivalent to “neither satisfied nor dissatisfied” and in NSG closer to “satisfied.”

**Table 1:** Average (standard deviation) of the variables with statistically significant differences between the Non-Segawa and Segawa groups for the WHOQOL-BREF Instrument Analysis performed with the Wilcoxon test adjusted for age and sex.

Variable	NSG (N = 34)	SG (N = 17)	p-value
Question 3	2.3 (1.04)	1.9 (0.78)	0.045
Question 4	2.4 (1.10)	3.2 (0.97)	0.022
Question 8	3.7 (0.81)	3.1 (1.03)	0.034
Question 19	3.7 (0.84)	3.1 (0.93)	0.049
Physical domain	68.5 (2.3)	59.2 (2.1)	0.036

Abbreviations: WHOQOL-BREF, World Health Organization Quality of Life – BREF instrument; NSG, Non-Segawa group; SG, Segawa group.

Additionally, the unadjusted Wilcoxon test indicated a difference for question 20, “How satisfied are you with your personal relationships?”, with a mean value of 3.5 in SG (“neither satisfied nor dissatisfied”) and 3.9 in NSG (“satisfied”), different at *p* = 0.029. However, the statistical significance decreased after correction for sex and age. This adjusted analysis also revealed a significant difference in the “Physical Domain” item of the questionnaire, scored on a scale of 0–100. These differences are summarized in Table 1.

### 3.2 Neuropsychiatric evaluation by MINI/MINI PLUS

Fisher's exact test showed statistically significant differences between the groups in the variables past major depressive episode (MDE PAST), generalized anxiety disorder (GAD), and attention-deficit/hyperactivity disorder in childhood (ADHD CHD) (see Table 2). For these last two variables, there was no occurrence in SG.

**Table 2:** Frequency and prevalence of neuropsychiatric disorders with statistically significant differences between the Non-Segawa and Segawa groups suggested by MINI/MINI PLUS.

Disorder	NSG (N = 21)	SG (N = 17)	p-value
MDE PAST	5 (23.8%)	10 (58.8%)	0.046
GAD	5 (23.8%)	0 (0%)	0.050
ADHD CHD	7 (33.3%)	0 (0%)	0.011

Abbreviations: MINI, Mini International Neuropsychiatric Interview; NSG, Non-Segawa group; SG, Segawa group; MDE PAST, Past major depressive episode; GAD, Generalized Anxiety Disorder; ADHD CHD, Attention deficit/hyperactivity disorder in childhood.

### 3.3 Evaluation of depression by the BDI – II

No significant differences between groups were observed concerning the affective component, physical component, and the gross score of the BDI-II test. Gross scores (13.24 for SG and 13.52 for NSG) meant borderline values between possible “no depression/minimal depression” (0−13) and possible “mild depression” (14–19). A contingency table of the gross scores suggested a prevalence of 41.2% for depressive symptoms (mild, moderate, or severe) in the SG vs. 42.9% in the NSG (Table 3).

**Table 3:** Frequency (percentage) for each category of the gross score used in the BDI - II inventory test.

Category	NSG (N = 21)	SG (N = 17)	Total
0–13 (No/Minimum depression)	12 (57.1%)	10 (58.8%)	22 (57.9%)
14–19 (Mild depression)	4 (19.1%)	2 (11.8%)	6 (15.8%)
20–28 (Moderate depression)	2 (9.5%)	5 (29.4%)	7 (18.4%)
29–63 (Severe depression)	3 (14.3%)	0 (0%)	3 (7.9%)

Abbreviations: BDI – II, Beck Depression Inventory – II; NSG, Non-Segawa group; SG Segawa group.

### 3.4 Cognitive evaluation using WMT-2

On a 0–18 scale, the mean gross score for the total sample was 10.8, with a mean percentile degree of 50.9 and an average performance rate (level III). The mean (s.d.) percentile was 52.7(33.2) for NSG and 48.6(27.6) for SG. No significant difference was observed in the scores obtained by WMT-2. In the NSG, 29.4% of the respondents presented a “below average” or “extremely low” performance rating (percentile ≤25) vs. 14.3% in SG.

### 3.5 Subjective assessment of daytime sleepiness

The mean gross score of the total sample in the ESS was 10.1, with a median of 11.0, and these values were considered borderline for an abnormal sleep score (> 10). The means(s.d.) for the evaluated variables did not vary significantly, including the gross score (10.3(4.8) for NSG and 9.8(4.0) for SG). The prevalence of abnormal sleep scales showing excessive daytime sleepiness in NSG and SG were 57.1% and 47.1%, respectively, and the participants were rated using the level of daytime sleepiness, according to Table 4.

**Table 4:** Frequency (percentage) for each category of daytime sleepiness used in ESS.

Daytime sleepiness category (gross score range)	NSG (N = 21)	SG (N = 17)	Total
Lower normal (0–5)	5 (23.8%)	3 (17.6%)	8 (21.1%)
Higher normal (6–10)	4 (19.1%)	6 (35.3%)	10 (26.3%)
Mild excessive (11−12)	7 (33.3%)	3 (17.7%)	10 (26.3%)
Moderate excessive (13–15)	2 (9.5%)	4 (23.5%)	6 (15.8%)
Severe excessive (16–24)	3 (14.3%)	1 (5.9%)	4 (10.5%)

Abbreviations: ESS, Epworth Sleepiness Scale; NSG, Non-Segawa group; SG Segawa group.

### 3.6 Sleep quality assessment

For the whole sample, the mean value for variable C1 (subjective quality of sleep) was 1.1, classifying self-assessed individuals as having a very good subjective quality of sleep. However, the overall mean PSQI score for all interviewed participants was 7.6 (7.7(4.7) in NSG vs. 7.4(3.0) in SG) on a 0–21 scale, which means that the interviewees self-rated themselves as having poor sleep quality (score > 5) in the month prior to their evaluation. 58.8% of GS participants and 82.4% of NSG participants were classified as “poor sleepers”.

### 3.7 Medication use and relationship with studied symptoms

The use of antidepressant drugs, as the selective serotonin reuptake inhibitors, occurred without statistically significant differences between the 34 NSG members and the 17 SG individuals (8 individuals in the NSG vs. 5 in the SG, *p* = 0.738), as well as the use of anxiolytics, like benzodiazepines (5 individuals in the NSG vs. 5 in the SG, *p* = 0.269) and the use of psychoactive drugs other than those mentioned, as biperiden, topiramate, melatonin, and pramipexole (4 individuals in the NSG vs. 4 in SG, *p* = 0.416).

Among the 17 SG members, 10 had motor symptoms (which would mean a penetrance of 58.8%), and 9 used L-Dopa for major motor symptoms (one patient not medicated with L-Dopa had a peripheral motor symptom, observed while washing dishes). In the SG, antidepressant drugs were used by 2 (22.2%) of the nine patients that used L-Dopa vs. 3 (37.5%) of the eight individuals that did not use L-Dopa (*p* = 0.620). None of the 34 individuals of the NSG used L-Dopa.

Finally, only 2 (11.8%) of the 17 SG individuals did not use L-Dopa or antidepressants or were diagnosed with a current, recurrent or past major depressive episode. Table 5 shows some statistical analyses of categorical variables related to depression based on the distribution of the participants into subgroups selected by type of informed psychoactive medication.

**Table 5:** Contingency of participants and *p*-value, by categorical variables of Major Depressive Episodes and some experiment groups, according MINI/MINI PLUS and medication use.

Categorical variable (with levels, N/Y:)	Experiment groups			p-value
MDE PAST		NON-LDOPA	LDOPA	0.436
	N	19	4	
	Y	10	5	
MDE PAST		NSG	SG NON-LDOPA	0.083
	N	16	3	
	Y	5	5	
MDE CURR		SG NON-LDOPA	SG LDOPA	0.082
	N	5	9	
	Y	3	0	
MDE CURR		NSG	SG NON-LDOPA	0.112
	N	19	5	
	Y	2	3	
MDE RECURR		NSG	SG NON-LDOPA	0.164
	N	17	4	
	Y	4	4	
MDE PAST		NSG	SG LDOPA	0.115
	N	16	4	
	Y	5	5	

Abbreviations: MINI, Mini International Neuropsychiatric Interview; N, No; Y, Yes; MDE PAST, Past major depressive episode; NON-LDOPA, Participants that did not use L-Dopa; LDOPA, Participants that used L-Dopa; NSG, Non-Segawa group; SG NON-LDOPA, Segawa group participants that did not use L-Dopa; MDE CURR, Current major depressive episode; MDE RECURR, Recurrent major depressive episode; SG LDOPA, Segawa group participants that used L-Dopa.

## 4 Discussion

The extension and severity of symptoms are pretty broad in individuals with *GCH1* variation. This variability indicates that other factors, including environmental, can modify the genetic action, influencing the expression of symptoms [26].

The availability of homogeneous samples is a problem in studying rare familiar diseases. Brüggemann et al. opted to study the NMS of autosomal dominant DYT/PARK-*GCH1* using non-familial case and control groups in order to reduce the influence of intrafamilial factors that were independent of GTP cyclohydrolase-1 deficiency and could affect the results [27]. In that work, Brüggemann et al. did not confirm the clinical impressions and biologically plausible assumptions of an increased frequency of NMS in DRD, although, as in most studies, the patients were on stable L-Dopa therapy [27].

In the present work, however, the intrafamilial pairing of the groups was chosen to provide to both groups the most similar family factors, genetic profiles (except on the specific studied *GCH1* variation), and environmental variables. Therefore, the differences in prevalence for the studied events would be more likely to be associated with the autosomal dominant DYT/PARK-*GCH1* variation.

The present study did not detect impairment of intellectual capacity in this DRD through the analysis of the WMT-2, which is different from the findings of López-Laso et al. [28]. Also, as stated by Furukawa (2019), although reports on the frequency of NMS in these individuals remain conflicting, in general, intellectual and cognitive function is typical in individuals with DYT/PARK-*GCH1* [12].

In the present work, statistically significant differences in the prevalence of some non-motor disorders or circumstances were found between the surveyed groups. Individuals with the genetic variation had in WHOQOL-BREF a worse perception of how safe they felt in their daily lives (*p* = 0.034) and less satisfaction with themselves (*p* = 0.049) and with their relationships (*p* = 0.029); in SG, a higher prevalence of past major depressive episodes in the MINI/MINI PLUS (58.8% in SG vs. 23.8%, *p* = 0.046) was detected, especially before the beginning of L-Dopa administration.

Data analysis of major depressive episodes (MDE) in Table 5, considering different subgroups based on the use of psychoactive medications, allows exciting discussions.

Comparing the frequency of MDE PAST between the subgroup of Segawa patients who used L-Dopa and the remaining group of all the participants who did not use this drug (including individuals from the NSG), no statistically significant difference was detected in the occurrence of MDE PAST (*p*-value 0.436), as detected when comparing the occurrence of MDE PAST between SG and NSG (p-value 0.046). This finding suggests that, for the evaluation of this categorical variable, it is not the use of L-Dopa but rather the genetic variation that is statistically associated with a higher occurrence of MDE PAST. Objectively, among 8 SG individuals (seven with no NMS), who were not using L-Dopa, 5 (62.5%) had MDE PAST detected by the analysis.

Even if not statistically significant, the comparison of the MDE PAST frequency between the SG individuals who did not use L-Dopa and the NSG individuals is also noteworthy (*p* = 0.083). In this case, only 5 of the 21 NSG individuals had MDE PAST, suggesting a possible association between genetic variation and MDE PAST.

Concerning current (CURR) and recurrent (RECURR) MDE, the analyses of these participant subgroups in Table 5 also provide a similar discussion, even though their *p*-values were slightly above 0.050, which may have been affected by the limited size of the available sample.

Comparing the frequency of CURR MDE between the SG individuals who did not use L-Dopa and the subgroup of individuals with the variation who used the drug, all 9 participants who used L-Dopa did not present criteria for CURR MDE. In contrast, 3 (37.5%) of the eight individuals with the genetic variation without using the drug received this diagnosis (*p* = 0.082). This association could suggest the possibility of a protective effect of L-Dopa replacement against CURR MDE in the studied individuals with the genetic variation.

Analyzing the frequencies of CURR and RECURR MDE between the SG individuals who did not use L-Dopa and NSG individuals (therefore, without the genetic variation), *p*-values were respectively 0.112 and 0.164. As the use of antidepressant drugs occurred without statistically significant differences between these groups, if sample sizes were more extensive, such analysis could even be closer to a statistically significant association, featuring a higher occurrence of these MDE with the genetic variation in the studied patients (50.0% of the SG members who did not use L-Dopa had RECURR MDE, vs. 19.0% of the NSG individuals). In this sense, it also corroborates the finding that comparing individuals from the NSG and the SG who used L-Dopa, the Fisher's Exact Test presented a p-value of 1 for the CURR and RECURR MDE variables, but a value of 0.115 for the MDE PAST (55.5% of the nine individuals with the variation that used L-Dopa had MDE PAST vs. 23.8% of the NSG individuals). This could suggest that L-Dopa replacement at low doses would be adequate for the control of motor symptoms and the approach of CURR and RECURR MDE in the studied individuals with genetic variation. The association could be plausible, as this supposed protective effect against depressive episodes was not observed in the MDE PAST analysis that refers to the period prior to L-Dopa use by individuals with autosomal dominant DYT/PARK-*GCH1*. This possibility should target future investigations, especially in other families with larger sample sizes.

Tadic et al. (2012) published a comprehensive review with results from 352 symptomatic DRD patients, heterozygous mutations in *GCH1*, and a pilot cohort of 23 outpatients [13]. Among 70 patients whose information on non-motor signs was obtained in the literature, 34% had depression, which also seemed high for those authors compared to the general population and even patients with Parkinson's disease [13]. In the pilot cohort study, 6 cases (32%) reported one or more non-motor signs, including depression and migraine, and all patients in that pilot study were treated with dopaminergic medication and reported NMS during treatment [13].

According to Antelmi's review on NMS in DYT/PARK-*GCH1*, neuropsychiatric features, and, particularly, depression, were reported in almost all available cohort studies [11,14,15,28]. As highlighted by Antelmi (2015), biochemical basis, together with the fact that sometimes they may precede motor signs or maybe the only clinical manifestation, would support that they are a primary feature [14,15]. Nevertheless, for Antelmi, some bias also needed to be considered, and future studies could be enlightening [11].

On the other hand, Timmers et al. (2017), after also highlighting that in DRD patients the occurrence of NMS is expected, found a higher lifetime prevalence of psychiatric disorders (61% vs. 29%, *p* < 0.05) and daytime sleepiness (11.2 vs. 5.7, p < 0.05) in a cohort of Dutch adults with DYT/PARK-*GCH1*, but not in children, compared to matched controls [16]. For those authors, in particular, the frequencies of generalized anxiety and agoraphobia were higher in patients with DRD (both 29% vs. 4%, p < 0.05), and adult patients had significantly lower scores on the mental component of the Health-Related Quality of Life Questionnaire than controls (47 vs. 54, p < 0.05), primarily associated with poorer sleep quality [16]. Data from 28 patients (18 adults and ten children from 10 families) contributed to that study, which suggested the possible involvement of serotonergic mechanisms [16].

The present study suggested in both groups a high prevalence of excessive daytime somnolence (47.1% for SG vs. 57.1%) and poor sleep quality, even slightly above the cut-off point for such classification (in 58.8% of SG vs. 82.4%), despite the high percentage of these individuals using L-Dopa for the dystonia and/or other psychoactive drugs. A high prevalence of current depressive symptoms in BDI-II also was detected in both groups (41.2% in SG vs. 42.9%). Nevertheless, generalized anxiety disorder (23.8%, *p* = 0.050) and attention-deficit/hyperactivity disorder in childhood (33.3%, *p* = 0.011) occurred exclusively in relatives without the variation. This finding can suggest more resilience to these disorders in the studied SG or a bias due to the greater use of psychoactive drugs in family members with the variation, although there is no statistically significant difference in this use, except L-Dopa in low dosages.

It is postulated that dopamine acts as an important facilitator of discriminative learning of the stimuli that represent safety and threat to individuals [29]. Therefore, genetic dopamine depletions, such as those determined in autosomal dominant DYT/PARK-*GCH1* and occurring prior to the individual's conditioning to the dopamine replacement stimulus, would be able to completely stop the acquisition of responses to conditioned threats [[30], [31], [32], [33]]. This would function as a “protective factor” or resilience for anxiety disorders. In addition, reducing the excitatory effects of dopamine in these individuals would be a potential justification for the lower prevalence of this disorder in the SG since reduced dopamine would also result in the reduction of adrenaline and noradrenaline, known as stimulants involved in anxiety.

A study by Del Campo et al. pointed out that the leading cause of Attention-Deficit/Hyperactivity Disorder may be related to the structural neuroanatomical differences in the reduced gray matter of the limbic and frontostriatal-cerebellar networks of the affected individuals and not to the altered availability of D2/D3 dopaminergic receptors or endogenous dopamine levels [34].

In the study of López-Laso et al. on neuropsychiatric symptoms of 14 outpatients with DRD from 2 families, impulsivity was detected exclusively in the seven adult individuals in the sample, regardless of L-Dopa replacement therapy, what happened in four of those seven [28].

By applying the adapted W component of MINI PLUS, all seven diagnosed childhood Attention-Deficit/Hyperactivity Disorder cases occurred in NSG individuals. As no SG participant had been treated with L-Dopa in childhood, it should be possible to postulate that low dopamine can protect the SG children against the Attention-Deficit/Hyperactivity Disorder, what was not seen in similar publication.

In an anterior study of this large family, considering only motor symptoms, the disease penetrance was 52.6% (10/19 patients) [17]; currently, the penetrance according to this criterion is 58.8%, as 10 SG patients had these symptoms in 17. However, as only 2 of the 17 SG members did not use L-Dopa for motor symptoms or antidepressants or were diagnosed with a current, recurrent, or past major depressive episode, it is suggested that autosomal dominant DYT/PARK-*GCH1* penetrance can indirectly reach 88.2% in this SG, considering the association of major depressive episode as part of the NMS minimally.

This study has limitations, and some biases can be postulated by selecting tools that may not be considered diagnostic, by the subjectivity of a self-assessment, and by the selection of intrafamilial groups. The limited sample size, characteristic of rare diseases, was also highlighted as a restraining factor. Additionally, for ethical reasons, the evaluations were carried out without interrupting the use of ongoing medications by the studied individuals.

Further studies are suggested with neurotransmitter dosages and clinical examination to investigate other evidence in these individuals. Considering the medication use and the simultaneity of different control groups from and outside the family, case-control studies with a more significant sample number could also be elucidative.

## 5 Conclusions

Due to the possibility that only NMS may occur in a family member affected by the *GCH1* variation, the recommendation to carry out diagnostic studies to investigate the variation in all the individuals of the family under risk is reinforced, especially in those who have no motor symptoms. Considering the association of at least a major depressive episode as part of the NMS, it is suggested that autosomal dominant DYT/PARK-*GCH1* penetrance may reach 88% in this family, compared to the prior penetrance estimated in 58.8%, considering only motor symptoms (10 in 17 individuals with the variant). Additionally, neuropsychiatric tests of all individuals with a molecular diagnosis in an affected family seem to be relevant to adequate clinical management.

## Ethical compliance statement

The Ethics Committee of the Federal University of Minas Gerais approved the study, under the number 06803312.8.0000.5149, performed between March 2016 and November 2017, following the ethical standards laid down in the 1964 Declaration of Helsinki and its later amendments.

Signed informed consent was obtained from all participants according to the approval of the Ethics Committee of the Federal University of Minas Gerais. Proof of informed consent is available upon request.

## Data access and responsibility

All research data are available at the Federal University of Minas Gerais archives upon reasonable request to the corresponding author. The correspondent author takes responsibility for the integrity of the data and the accuracy of the data analysis.

## Authors' contributions

All co-authors have been substantially involved in the study and/or preparation of the manuscript. Each author (by his/her initials) is listed beneath this information, and his/her specific roles in the project and manuscript preparations are identified by number and letter.

1. Research project: A. Conception, B. Organization, C. Execution (Data Collection);

2. Statistical Analysis: A. Design, B. Execution, C. Review and Critique;

3. Manuscript Preparation: A. Writing of the first draft, B. Review and Critique;

ACAJ: 1A, 1B, 1C, 2A, 2B, 2C, 3A, 3B; MVD: 1A, 1C, 3A, 3B; AMCM: 1A, 2A, 2B, 2C, 3A, 3B; ASL: 1C, 2B, 3B; DCVN: 1A, 3B; ERV: 1A, 1B, 1C, 2A, 2B, 2C, 3A, 3B.

All authors read and approved the final manuscript.

## Disclosures

Funding Agency and Conflict of Interest:

This work was partly supported by the Laboratory of Inborn Errors of Metabolism of the School of Medicine of the 10.13039/501100007374Federal University of Minas Gerais – Brazil. Dr. Valadares was the chief of this laboratory during the time of this research. Other sponsors have not influenced the content of the article. The supporting source had no role in the study design, collection, analysis, and interpretation of data, [[1], [2], [3], [4], [5], [6], [7], [8], [9], [10], [11], [12], [13], [14], [15], [16], [17], [18], [19], [20], [21], [22], [23], [24], [25], [26], [27], [28], [29], [30], [31], [32], [33], [34]] writing of the report, and any involvement or restrictions regarding the submission of the report for publication. The authors declare no conflicts of interest relevant to this work.
